# Addressing vaccination hesitancy in Europe: a case study in state–society relations

**DOI:** 10.1093/eurpub/cky155

**Published:** 2018-11-01

**Authors:** Katharina Kieslich

**Affiliations:** Department of Political Science, University of Vienna, Vienna, Austria

## Abstract

In light of recent outbreaks of diseases such as measles in Europe, policymakers and public health practitioners are seeking strategies to address anti-vaccination attitudes and to increase immunization rates. Identifying effective strategies that will not further alienate vaccination sceptics raises challenges that go to the heart of relations between the state and society. Drawing on accounts of state–society relations, this article discusses how the problem of vaccination hesitancy might be explained from a political science perspective. Discourse-analytical approaches emphasize the importance of storylines, politics and social context in explaining a range of phenomena. Given the number and strength of prevailing discourses in groups with anti-vaccination sentiments, the literature on discourse coalitions can offer perspectives on the challenges that arise in designing strategies to address vaccine hesitancy. Paying closer attention to individual reasons why parents are vaccine hesitant might allow for designing strategies that are more suited to address concerns. However, given the pervasiveness of the discourses of anti-vaccination movements, challenges in reaching citizens who are sceptical of vaccines will remain.

## Introduction

Theories and research on state–society relations seek to analyze the political patterns and behaviours that emerge from interactions between state entities and societal groups. Work on state–society relations ranges from studies on the influence of interest groups on policymaking to the role of non-governmental organizations (NGOs) and civil society on political processes in Africa and Asia.^1^ It offers a lens for understanding a range of complex issues, including issues faced by public health practitioners and policymakers. One of these issues is a concern over decreasing vaccination rates and increasing vaccination hesitancy, which this paper explores as a case study in state–society relations. Drawing on the notion of discourse coalitions it will show that state–society relations in vaccination policy are characterized by contestation over the credibility of scientific evidence and a distrust of citizens in public health institutions.

In June 2017 a group of parents in Northern Italy announced that they would seek asylum in Austria in reaction to the introduction of mandatory childhood vaccinations against 12 diseases in Italy.^2^ The introduction of mandatory requirements was a policy response by the Italian government to a rise in outbreaks of preventable illnesses such as measles. The ensuing reactions of the group of parents represents the latest escalation of long-standing disagreements between governments and groups of citizens over the right approach to vaccination policy, with Italy not the only country to change vaccination requirements. Countries such as Germany and France have followed suit to address concerns over decreasing vaccination rates. Vaccination hesitancy was identified as one of the main drivers of falling vaccination coverage in the European Commission’s recent recommendations to strengthen European Union cooperation on vaccine-preventable diseases.^3^

The complex reasons why parents hesitate to, or choose not to, have their children vaccinated are now well-evidenced.^4–6^ However, the underlying dynamics of the relationship between governments and its citizens that help explain why finding appropriate, and effective, policy responses can be challenging, are documented less well. Insights from political science can help deepen our understanding of the challenges.

## State–society relations in political science

Accounts of state–society relations represent core issues and questions with which political scientists have been concerned since the emergence of the discipline. Given its name, it will come as no surprise that scholars of state–society relations focus on the relationships, the interplay of, and the interdependence between state and society in different countries.^7^ However, this is where the clarity ends, and the complexity begins. The conceptualizations of state and society, and the demarcation between the state and society, are some of the issues with which scholars have grappled.^8^ From this, a trend has emerged that emphasizes complexity and interdependence in relations between state actors and society actors. At its core, the field is concerned with the influence that these actors have on each other, and the conditions under which they are successful in shaping policy.

Early scholarship on relations between state and society, influenced by Max Weber, conceptualized the state as a unitary, autonomous actor that exerts authority over members of society through administrative rules and the law.^7^ In these accounts, policymaking is seen a top-down process in which government bureaucrats hold much power due to resources such as access to technical knowledge and policymakers. Societal groups and citizens are seen as mostly passive actors in a system that is skewed towards government resources and power. Over the years, this view has given way to pluralist, bottom-up understandings of state–society relations in which interest groups, social movements, corporations and developments at international, national and federal levels are perceived to have an effect on how policy is made and how policy priorities are set.^7^ The relevance to vaccination scepticism of these articulations is reflected in the fact that societal groupings in which anti-vaccination sentiments are prominent are frequently referred to as anti-vaccination movements.

The current prevalent understanding of state–society relations is to view relations as nuanced, complex and interdependent processes in which the distinctions between state actors such as ministers and bureaucrats, and society actors such as interest groups and grassroots movements, are viewed as unhelpful in understanding the complex dynamics of policymaking. Sabatier’s Advocacy Coalition Framework, e.g. posits that it is more useful to think of policymaking as a process in which state and society actors form alliances, so-called advocacy coalitions, around issues or belief systems rather than around the membership of a category of state or society actors.^9^^,^^10^ The discourse coalition approach, applied to the case of vaccination hesitancy in a later section, on the other hand uses the notion of storylines to illustrate the importance of the interpretation of issues in policymaking and policy change.^11^ Policymaking is seen as a constant process of argumentation to define and redefine issues in terms of storylines that are conceptualized as ‘[…] symbolically constructed discursive structures […]’.^11^ These storylines act as powerful narratives which hold coalitions, and the belief systems of their members, together without necessitating any formal coordination of political activity. Such storylines, or the way the narratives around issues are constructed, are an influential variable as they can be largely resistant to the presentation of facts and evidence.

## Discussion

### State–society relations, public health and vaccination hesitancy

The field of state–society relations comprises not one approach, but multiple lenses that ascribe varying degrees of policymaking influence to state actors and society actors, respectively. Through these lenses the public health community can variously be conceived as an interest group, i.e. a group seeking to influence policy on a commonly held goal, or an epistemic community,^12^ i.e. a group of experts who helps policymakers to identify policy problems and solutions through the application of knowledge and expertise on technical subjects. Depending on their role in government or on scientific panels, public health experts might also be viewed as an instrument of the state. These categorizations are neither static nor impermeable, and in discussing its role in the policymaking process attention needs to be paid to the fact that the public health community in Europe and elsewhere is not a uniform, monolithic actor. Depending on their positions, members of the public health community hold responsibilities as employees in health ministries, local public health authorities, professional associations or research groups.

Similarly, research has shown that individuals and groups with anti-vaccine sentiments cannot be conceptualized as a homogeneous group, and that reasons for vaccination scepticism are varied.^13^ However, the heterogeneity of vaccination sceptics is distinct from the heterogeneity of the public health community in one important respect: regardless of the ontological lens one employs, it is difficult to argue that current anti-vaccination groupings are perceived by policymakers and the public health community as anything other than a societal force that needs to be contained. The power and influence of vaccination sceptics lies in the fact that their action, or rather inaction to vaccinate children, has a direct effect on the public health goals of state or health authorities by undermining them. Not only that, but the inaction to vaccinate also affects other members of society by decreasing the chances of achieving herd immunity in a population and endangering those who cannot get vaccinated due to allergies to vaccine components, for example. Vaccination scepticism represents an interesting case study of state–society relations as the power of vaccination sceptics stems largely from an inaction to partake in a recommended, or mandatory, public health intervention without the need to be organized in formalized coalitions or interest groups; this is also what distinguishes anti-vaccination groupings from other social movements.

Rather than the threat of mass mobilization to affect policy change, a common identifier of social movements,^14^ the most powerful resource at the disposal of vaccination sceptics is their ability to undermine public health goals. Ironically, this frequently leads to the introduction of policies such as mandatory vaccination programmes that are diametrically opposed to what sceptics believe in. They have a role in the policymaking process inasmuch as that their convictions are used by policymakers to justify policies that are contrary to the beliefs espoused by the groups. However, recent developments around the world suggest that this might be changing, with populist political movements employing the anti-vaccination rhetoric for political gain, a phenomenon that is returned to in a later section.

### Designing policies to address anti-vaccination sentiments

Common to the approaches on state–society relations is a concern with the locus of authority, influence and responsibility in policymaking. A well-known challenge facing government and public health authorities is designing tailored strategies that successfully reach vaccination sceptics. Reaching an audience or a part of a country’s population requires an understanding of the space that this audience occupies. In the case of vaccination hesitancy, the space where sceptics are found might be parent communities in schools or kindergartens, for example. The link between target audiences and policies becomes more complicated when one thinks about whether vaccination sceptics might be found in paediatric surgeries, considering that variants of vaccination scepticism might exhibit a general scepticism of the medical profession.^14^^,^^15^ The difficulty in defining a policy audience is further exacerbated by the prolific space that the internet offers for anti-vaccination groups to spread their beliefs and to gain followers.

The effect of the internet on state–society relations is gaining traction as a field of study.^16^ Here, scholars are preoccupied with the question of how state and society actors benefit from the internet as a vehicle to promote their goals. The context and characteristics of the political system in a given country plays a role in the extent to which state and society actors benefit from the use of the internet, with societal actors in liberal democratic systems benefitting from the opportunities it offers for engagement and reach, whereas access to the internet might be restricted in other political systems.^16^ As the use of the internet and social media channels spreads, the challenge of vaccination hesitancy presents itself as an increasingly transnational problem, a characteristic that further complicates policymakers’ tasks of designing effective policies.

Undoubtedly the internet provides a valuable tool for those sceptical of vaccination, and several studies have shown that key messages are similar across websites.^15^^,^^17^ The groups, individuals and websites promoting anti-vaccination sentiments are not always affiliated with an established organization or movement. While more formalized structures such as the French National League for Liberty in Vaccination in France or the parent initiative ‘Ökokinderrechte Südtirol’ in Northern Italy exist, the groups behind websites such as ‘www.impfen-nein-danke.de’ (vaccination no thank you) in Germany are more difficult to identify. This is problematic for two reasons. First, it makes it difficult to identify groups or individuals with whom to talk if state or health authorities wanted to engage in a dialogue. Second, it makes it difficult to assess how widespread or serious the traction of messages is. However, the prevailing discourses on websites are similar and include fears over the safety and effectiveness of vaccines, advocacy for alternative and natural approaches to health, a concern about safeguarding civil liberties such as parental autonomy, and conspiracy theories about the links between pharmaceutical companies, governments and the medical profession.^15^

These discourses speak to the importance of storylines that discourse-analytical approaches such as the notion of discourse coalitions emphasize.^10^ Vaccination sceptics can be conceptualized as informal coalitions of individuals or groups who find their personal beliefs reflected in the storylines promoted in the anti-vaccination discourse. The storylines are characterized by a scepticism about the credibility of scientific evidence and a distrust in the authorities, government or otherwise, who are the disseminators or producers of scientific knowledge. In her research on the litigation cases of the alleged connection between autism and the MMR vaccine, Kirkland highlights the pervasiveness of the distrust in scientific evidence of vaccination sceptics: ‘Scientific evidence, no matter how clear it seems to be to the people who produce it […], does not have magical power to change minds’.^18^ The case explored by Kirkland also shows that problems can be redefined to fit new storylines; when the scientific merits of the case of the anti-vaccination plaintiffs were discredited in court, the narrative was reinvented to tell the story of governmental institutions using all their power to avoid a loss against a small and powerless entity such as the plaintiffs.

The variety of beliefs and messages promoted by anti-vaccination groups poses problems for designing effective policy strategies. Each reason for choosing not to vaccinate children in theory requires its own tailored policy response. This argument is based on a branch of political science that argues that characteristics of a problem or policy area determine political conflicts and policy options, in Lowi’s words ‘policy shapes politics’.^19^^,^^20^ For state–society relationships this suggests that relations differ in different policy sectors.^7^ This explains why some policy problems such as vaccination scepticism seem intractable, and persist over years and even decades. The more contested a policy area, the more difficult it is to design policies that achieve what is intended. To address the concern about the safety of vaccines, education campaigns might indeed be appropriate although the evidence for their effectiveness is uncertain.^21^ The introduction of mandatory vaccinations on the other hand might result in an inevitable backlash that can lead to policy failure if anti-vaccination groups are worried about the interference of the state in the right of parents to decide what is best for children. The concern about the safety of vaccines is of a different nature than the concern about the interference of the state in parental autonomy.

The role of the characteristics of a policy area, and the ensuing effect on state–society relations, goes a long way in helping to explain the difficulty in designing effective policy responses to vaccination hesitancy. The area is characterized by discourse coalitions, multiple and not easily identified locations of social activity and the lack of a unified agenda of anti-vaccination activists. Moreover, recent political developments suggest that the containment of anti-vaccination voices to non-mainstream public platforms might be changing, which is likely to have an effect on state–society relations. Increasingly, anti-establishment parties in Europe such as the 5Star Movement in Italy are putting the question of mandatory vaccination programmes on their political agendas, although, in case of the 5Star Movement its opposition is not against vaccines *per se*, but against the state-mandated vaccination requirement.^22^ The danger is that over time vaccine sceptics might gain a political platform that elevates their cause and legitimates their views.

What is evident from anti-establishment movements employing the anti-vaccination discourse for their gain, as well as from countries introducing revamped vaccination programmes, is that much of the political debate focuses on who should be responsible for (i) deciding whether children should be vaccinated, and (ii) implementing vaccination programmes. From a political science perspective, these questions raise long-standing issues about the role of the state in intervening in people’s lives. It might be tempting to brush aside concerns of vaccination sceptics as invalidated and dangerous, but they raise issues with which scholars of political theory and philosophy, and constitutional theory have grappled with for a long time. A detailed exploration of this literature is beyond the scope of this article, suffice it to say that questions of balancing parental autonomy and the autonomy over one’s own body with public interests such as maintaining or achieving herd immunity will always be subject to a degree of contestation and deliberation in liberal democracies.

The ongoing debate over the appropriate balance between coercion and autonomy in public health and other areas of state activity is one of the reasons why we are seeing divergent policy approaches to the same problem of decreasing vaccination rates across Europe as countries seek to ensure that national and local state–society relationships are not undermined. For example, German policymakers changed the law in 2015 to introduce a mandatory vaccination consultation for parents before their children start school.^23^ Public health authorities can fine parents up to 2500 Euros if parents consistently resist to attend a consultation. In 2017 the statutory obligations were further refined to introduce a clause that mandates nurseries to notify public health authorities if parents fail to show a written confirmation that they have attended the consultation.

The consultation requirements notwithstanding, the choice for or against vaccinations still lies with the parents. This can be explained with reference to Germany’s health care system that is corporatist, self-governed and federalized at its core. Any state-mandated vaccination programmes are likely to be met with scepticism not just from parts of the population, but also from the self-governing health care organizations such as the statutory sickness funds and the professional doctors’ associations who are used to negotiate service provision and funding amongst themselves.

Despite the comparative lack of more punitive measures such as the refusal of a kindergarten place if a child is not vaccinated, the changes in the law met some opposition in Germany. Criticism centred mainly on the question of who should implement measures on vaccination. Critics argued that putting the onus of reporting parents who refuse vaccination consultations on kindergarten head teachers and staff not only increases their administrative burdens, but also undermines the relationship between parents and staff.^24^ The debates also focussed on the locus of public health responsibilities, with critics arguing that education institutions should not be burdened with public health tasks. This illustrates the complexities of state–society relations in that even so-called state actors will not always agree on their responsibilities for public health.

## Conclusion

The attempt to explain some of the challenges that are involved in seeking policy solutions to vaccination hesitancy raises the question of where this leaves the public health and the policymaking community with regard to the available options for increasing vaccination rates. The following proposals are intended to contribute to a self-critical debate about how we might open the space to more tailored policy solutions.

First, it is important to evaluate our own ambiguities as a public health community. For example, as a scientific community we acknowledge that the interpretation of evidence is not always straightforward, leading to uncertainty in some cases. Yet, we struggle to acknowledge parents’ concerns over uncertainty in vaccination. This is because the comparative effectiveness and positive impact of large-scale vaccination programmes is a scientifically proven accomplishment of modern medicine. However, this accomplishment does not negate the fact that the quest for certainty in science is ongoing, and part of this quest involves engaging with individuals or groups who are not convinced of the evidence they are presented. Similarly, acknowledging mutual concerns about the role of the pharmaceutical industry, short of conspiracy theories, might lead to a more constructive dialogue. Finally, we must ask how to resolve the perceived tension between the advocacy of patient choice in health care on the one hand, and the more punitive measures in vaccination policy if parents decide against vaccinating their child on the other.^14^

For policymakers, paying closer attention to individual reasons why parents are vaccine hesitant might allow for designing strategies that are more suited to address the characteristics of the given concerns, much in line with Lowi’s observations.^19^^,^^20^ Prerequisites for the success of vaccination policies are an end to the fight over the locus of authority for their implementation, and the acceptance of joint responsibility for public health by coalitions of actors who are concerned with declining immunization rates. Vaccination policy is no different from other areas in public health; to be successful, actors at all levels, across governmental and non-governmental institutions, must work together to achieve goals. In doing so, it is crucial to recognize the storylines that make anti-vaccination discourses powerful, and to embrace the possibility of moving away from policy narratives that are focussed solely on factual information to ones that are interspersed with personal stories to which people might relate and connect.

## References

[1] Mercer C Reconceptualizing state-society relations in Tanzania: are NGOs ‘making a difference’? Area 1999;31:247–58.

[2] BBC. Parents Eye Austrian Asylum in Italy Vaccination Dispute. BBC News (14 March 2018, date last accessed).

[3] European Commission. Council Recommendation on Strengthened Cooperation against Vaccine Preventable Diseases. Eur-Lex. Available at: https://eur-lex.europa.eu/legal-content/EN/TXT/? uri=COM%3A2018%3A244%3AFIN (17 July 2018, date last accessed).

[4] Hobson-West P ‘Trusting blindly can be the biggest risk of all’: organised resistance to childhood vaccination in the UK. Sociol Health Illn 2007;29:198–215. 17381813 10.1111/j.1467-9566.2007.00544.x

[5] Kirkland A The legitimacy of vaccine critics: what is left after the autism hypothesis? J Health Polit Policy Law 2012;37:69–97. 22003097 10.1215/03616878-1496020

[6] Colgrove J, Abiola S, Mello MM HPV vaccination mandates – lawmaking amid political and scientific controversy. N Engl J Med 2010;363:785–91. 20818883 10.1056/NEJMsr1003547

[7] Sellers JM State-society relations In: Bevir M, editor. The SAGE Book of Governance. London: SAGE Publications, 2011: 124–42.

[8] Mitchell T The limits of the state: beyond statist approaches and their critics. Am Pol Sci Rev 1991;85:77–96.

[9] Sabatier PA, Weible CM The advocacy coalition framework: innovations and clarifications In: Sabatier PA, editor. Theories of the Policy Process. Boulder: Westview Press, 2007: 189–223.

[10] Sabatier PA The advocacy coalition framework: revisions and relevance for Europe. J Eur Public Policy 1998;5:98–130.

[11] Fischer F Reframing Public Policy: Discursive Politics and Deliberative Practices. Oxford: Oxford University Press, 2003: 102.

[12] Haas PM Introduction: epistemic communities and international policy coordination. Int Organ 1992;46:1–35.

[13] Luyten J, Desmet P, Dorgali V, et al Kicking against the pricks: vaccine sceptics have a different social orientation. Eur J Public Health 2014;24:310–4. 23813706 10.1093/eurpub/ckt080

[14] Blume S Anti-vaccination movements and their interpretations. Soc Sci Med 2006;62:628–42. 16039769 10.1016/j.socscimed.2005.06.020

[15] Kata A A postmodern Pandora’s box: anti-vaccination misinformation on the Internet. Vaccine 2010;28:1709–16. 20045099 10.1016/j.vaccine.2009.12.022

[16] Drezner DW Weighing the scales: the internet’s effect on State-Society Relations. Brown J World Aff 2010;16:31–44.

[17] Song MYJ, Abelson J Public engagement and policy entrepreneurship on social media in the time of anti-vaccination movements In: Adria M, Mao Y, editors. Handbook of Research on Citizen Engagement and Public Participation in the Era of New Media. Hershey: IGI Global, 2017: 38–57.

[18] Kirkland A Credibility battles in the autism litigation. Soc Stud Sci 2012;42:237–55. 22848999 10.1177/0306312711435832

[19] Lowi TJ American business, public policy, case-studies and political theory. World Politics 1964;16:677–715.

[20] Lowi TJ Four systems of policy, politics, and choice. Public Adm Rev 1972;32:298–310.

[21] Dubé E, Gagnon D, MacDonald NE; SAGE Working Group on Vaccine Hesitancy. Strategies intended to address vaccine hesitancy: review of published reviews. Vaccine 2015;33:4191–203. 25896385 10.1016/j.vaccine.2015.04.041

[22] Wheaton S, Zampano G Vaccine Debate Gives Italian Election Campaign a Shot in the Arm. Politico. Available at: https://www.politico.eu/article/vaccine-debate-gives-italian-election-campaign-a-shot-in-the-arm/ (12 March 2018, date last accessed).

[23] Aerzteblatt. Kindergardens to report parents in case of missing vaccination consultation in the future. [Kitas sollen Eltern bei fehlender Impfberatung künftig zwingend melden]. Aerzteblatt News. Available at: https://www.aerzteblatt.de/nachrichten/76834/Kitas-sollen-Eltern-bei-fehlender-Impfberatung-kuenftig-zwingend-melden (12 March 2018, date last accessed).

[24] Becker KB Compulsory reporting for Kindergardens. [Meldepflicht für Kitas]. Sueddeutsche Zeitung Available at: http://www.sueddeutsche.de/politik/impfungen-meldepflicht-fuer-kitas-1.3522495 (12 March 2018, date last accessed).

